# Association between free triiodothyronine and diabetic retinopathy: insights from a longitudinal cohort study and Mendelian randomization

**DOI:** 10.3389/fendo.2025.1677122

**Published:** 2025-12-17

**Authors:** Xiaotong Feng, Hongling Zhao, Yongsong Xu, Xianhua Li, Haodi Cao, Yingxiang Wang, Dong Zhao, Jing Ke

**Affiliations:** 1 Center for Endocrine Metabolism and Immune Diseases, Beijing Luhe Hospital, Capital Medical University, Beijing, China; 2 Laboratory for Clinical Medicine, Capital Medical University, Beijing, China

**Keywords:** non-proliferative diabetic retinopathy, proliferative diabetic retinopathy, thyroid hormones, free triiodothyronine, Mendelian randomization

## Abstract

**Background:**

Growing evidence indicates that thyroid function plays a critical pathophysiological role in diabetic microvascular complications. Nevertheless, the specific association between thyroid hormones, particularly free triiodothyronine (fT3), and diabetic retinopathy (DR) remains controversial.

**Methods:**

After applying the inclusion and exclusion criteria, 3703 patients were included in the baseline analysis. Multivariate logistic regression models were employed to assess the cross-sectional association between baseline fT3 levels and both the prevalence of DR. Subsequently, 1476 patients who underwent follow-up fundus photography were eligible for the retrospective cohort study. This secondary analysis examined the relationship between baseline fT3 quartiles and the risk of DR onset or progression. Additionally, two-sample Mendelian randomization (MR) analysis was performed to analyze the causal effect of circulating fT3 on non-proliferative DR (NPDR) and proliferative DR (PDR).

**Results:**

In the cross-sectional analysis, higher fT3 levels were inversely associated with DR after multivariable adjustment, including NPDR (OR = 0.61, 95% CI: 0.50-0.74) and PDR (OR = 0.24, 95% CI: 0.13-0.44). In the longitudinal cohort, patients with moderate fT3 levels (Q2–Q3) had a lower risk of DR onset or progression versus the lowest quartile (Q1). This protective association remained significant in those with suboptimal glycemic control (HbA1c ≥7%) or longer diabetes duration (≥ 10 years), with risk reductions of 43% (Q2) and 37% (Q3) in the former, and 44% (Q2) and 49% (Q3) in the latter. Notably, among older patients (≥ 55 years), the benefit extended across Q2-Q4. Finally, the MR analysis suggested a potential protective effect of higher fT3 levels on NPDR (MR Egger, OR = 0.131, 95% CI: 0.023-0.755, *P* = 0.044).

**Conclusion:**

In conclusion, our study demonstrated an inverse association between fT3 levels and the risk of both NPDR and PDR. Moderate fT3 levels were associated with a lower risk of DR onset or progression, particularly among patients with suboptimal glycemic control (HbA1c ≥7%) or longer diabetes duration (≥ 10 years). In older patients (≥ 55years), even relatively higher fT3 levels may be protective. MR analysis suggested a potential protective effect of elevated fT3 levels on the risk of NPDR, which was significant only in the MR Egger model.

## 1 Introduction

Diabetic retinopathy (DR) is a leading cause of visual impairment and blindness among working-age adults, affecting approximately one-third of individuals with diabetes mellitus (DM) (1, 2). As the global prevalence of DM is estimated to rise to 629 million by 2045, the burden of DR is expected to increase accordingly (3). According to the International Clinical Diabetic Retinopathy Disease Severity Scale, DR is broadly categorized into two stages: non-proliferative DR (NPDR), proliferative DR (PDR). The hallmark features of NPDR include microaneurysms, venous beads and capillary dilation, which impair blood flow and compromise retinal integrity. These changes can lead to diabetic macular edema, a major cause of vision loss (4). PDR, the advanced stage of DR, is characterized by neovascularization, vitreous hemorrhage, and retinal detachment, posing a serious threat to vision (1). The progression of DR is driven by multiple risk factors such as systolic blood pressure, renal dysfunction and so on, all of which substantially impair patients’ quality of life (5).

Thyroid hormones, as central regulators of glucose and lipid metabolism, are increasingly recognized as a potential contributor to DM pathophysiology (6–8). Notably, the prevalence of hypothyroidism among individuals with T2DM range from 6% to 20% across ethnic groups, suggesting bidirectional thyroid function and DM (7). Beyond overt dysfunction, even variations within the euthyroid range are relevant, lower free triiodothyronine (fT3) levels has been associated with increased risk of DM (9).

Emerging evidence suggests a potential role of thyroid dysfunction in the development of diabetic microvascular complications. In T2DM patients, lower fT3 levels independently predict the progression of diabetic nephropathy (10, 11). Some cross-sectional studies have reported inverse associations between fT3 levels and DR prevalence-even within the normal thyroid function range (12, 13). Others, including a mendelian randomization (MR) analysis found no causal relationship (14). Importantly, all existing studies to date are cross-sectional or genetically inferred, lacking longitudinal evidence to determine whether thyroid dysfunction contribute to DR pathogenesis or merely reflects concurrent metabolic disturbances.

To address this gap, we conducted a dual-phase study comprising a cross-sectional analysis to evaluate the association between fT3 levels and the severity of DR severity, as well as a longitudinal cohort to assess baseline fT3 levels predict the onset or progression of DR in patients with DM. Moreover, we performed a two-sample MR analysis to test the causal hypothesis, leveraging summary statistics from large-scale GWAS. Our findings aim to clarify the relationship between thyroid function, and to provide a potential basis for risk stratification and early screening strategies in clinical practice.

## 2 Methods

### 2.1 Study design and population

The clinical study combined a cross-sectional analysis with a retrospective cohort design. We enrolled diabetic patients from the National Metabolic Management Center (MMC) at Beijing Luhe Hospital between October 2017 and October 2022. The diagnosis of DM was based on the criteria established by the American Diabetes Association (15). To ensure data quality and the relevance of clinical endpoints, we applied several exclusion criteria: (1) Patients without thyroid function testing at their initial visit were also excluded. (2) Individuals with a history of thyroid cancer, hyperthyroidism, or subclinical hyperthyroidism were not included in the analysis. (3) For ophthalmologic assessment, participants were excluded if baseline fundus photographs were not available or if ocular conditions (e.g., cataracts) interfered with the grading of DR. A detailed flow chart illustrating the patient selection process is provided in **Supplementary Figure 1**.

### 2.2 Data collection

Data collection followed the standard protocol of MMC (16, 17). Demographics (age, sex), lifestyle (smoking history, drinking history), history of medication and clinical parameters (DM duration, weight, height, waist circumference, and hip circumference) were recorded. Body mass index (BMI) was calculated as weight (kg) divided by height squared (m²). Blood samples were analyzed for glycosylated hemoglobin (HbA1c), fasting blood glucose (FBG), fasting insulin, serum triglyceride (TG), total cholesterol (TC), low-density lipoprotein (LDL-C), and high-density lipoprotein (HDL-C). Thyroid function indicators (fT3, fT4, TSH) were detected by electrochemiluminescence immunoassays using an Abbott Architect I2000 (Abbott Diagnostics, Abbott Park, IL, USA). DR was assessed via digital fundus photography, and images were independently graded by two ophthalmologists blinded to clinical information, according to the International Clinical Diabetic Retinopathy Severity Scale, with specific criteria as follows: (1) Mild NPDR: Fundus photography shows only microaneurysms, without other retinal lesions; (2) Moderate NPDR: Fundus photography reveals microaneurysms, accompanied by possible retinal hemorrhages, hard exudates, and/or cotton wool spots. The severity of lesions does not meet the diagnostic criteria for severe NPDR; (3) Severe NPDR: Fundus photography is characterized by one or more of the following features: a. more than 20 intraretinal hemorrhages in each of 4 retinal quadrants; b. prominent venous beading in ≥2 quadrants; c. significant intraretinal microvascular abnormalities (IRMA) in ≥1 quadrant. No signs of proliferative lesions are observed; (4) PDR: Fundus photography demonstrates the presence of neovascularization (on the disc or elsewhere), vitreous hemorrhage, and/or preretinal hemorrhage. In addition, cases with insufficient image quality were excluded to avoid misdiagnosis. In the cross-sectional phase, DR was graded at baseline. In the cohort phase, patients without DR at baseline were followed for DR onset, while patients with baseline DR were monitored for progression.

### 2.3 Statistical analysis

Clinical data were analyzed using SPSS 29.0. Continuous variables were presented as mean ± standard deviation (SD) for normally distributed data, or as median with interquartile range (IQR) for skewed data. Categorical variables were presented as frequencies and percentages Differences among multiple groups were assessed using one-way ANOVA, nonparametric tests, or chi-square tests, as appropriate. Multivariate logistic regression analysis was performed to identify independent risk factors for NPDR and PDR in the cross-sectional study. In the retrospective cohort study, patients were stratified by baseline fT3 quartiles (Q1: ≤ 2.70 pg/ml, Q2: 2.70 pg/ml-2.98 pg/ml, Q3: 2.98- 3.27 pg/ml, Q4: > 3.27 pg/ml). The association between fT3 quartiles and the risk of DR onset or progression was evaluated using multivariate logistic regression. A *P*-value less than 0.05 was considered statistically significant.

The two-sample MR analysis was conducted to investigate the causal effect of circulating fT3 on NPDR and PDR. Instrumental variables (IVs) were selected based on three stringent criteria: (1) genome-wide significance (*P* < 5×10^−8^), (2) genetic independence (pairwise linkage disequilibrium r² < 0.001 within 10,000 kb clumping windows), and (3) sufficient instrumental strength: F-statistic > 10, calculated as


$$F=\frac{(N-2)\times R^{2}}{1-R^{2}}$$

where N is the sample size of the fT3 GWAS dataset, and $R^{2}$ is the proportion of fT3 variance explained by each SNP. Fourteen independent SNPs strongly associated with fT3 were selected as instrumental variables from GWAS data (18). Summary statistics for NPDR and PDR outcomes were obtained from the FinnGen Consortium (Release R9), accessed through the IEU OpenGWAS database (https://gwas.mrcieu.ac.uk/), and the following SNP were used: NPDR (finn-b-DM_BCKGRND_RETINA_NONPROLIF) and PDR (finn-b-H7_RETINOPATHYDIAB_PROLIF). To ensure accurate allele harmonization, we excluded palindromic SNPs, including rs11626434, as these variants are prone to strand ambiguity in effect estimation.

Causal estimates were derived using multiple MR methods including inverse variance weighted (IVW), MR-Egger regression, weighted median, simple mode, and weighted mode. MR-Egger intercept and Cochran’s Q tests were performed to evaluate horizontal pleiotropy and heterogeneity. To evaluate the robustness of our findings, a series of sensitivity analyses were performed. These included tests for horizontal pleiotropy and heterogeneity, as well as a leave-one-out analysis. Furthermore, the results were visualized using scatter plots, forest plots, and funnel plots. Analyses were implemented in R (R version 4.4.1) using the TwoSampleMR package, with significance set at *P* < 0.05.

## 3 Results

### 3.1 Baseline characteristics of participants in the cross-sectional study

After applying the inclusion and exclusion criteria, 3703 patients were included in the cross-sectional analysis. Among enrolled patients, 2630 (71.0%) had no DR (DM group), 1018 (27.5%) had NPDR, and 55 (1.5%) had PDR. Demographic and metabolic characteristics were shown in **Table 1**. Patients in NPDR (52.0 ± 11.8 years) and PDR patients (53.4 ± 10.7 years) were older than patients in DM group (50.2 ± 12.9 years). The proportion of patients with DM duration exceeding 10 years progressively increased across groups (DM: 23.6%, NPDR: 47.9%, PDR: 60.0%, *P* < 0.001). Suboptimal glycemic control (HbA1c ≥7%) was more frequently observed in patients with NPDR (88.5%) and PDR (85.2%) compared to DM group (72.0%). This finding was consistent with the elevated levels of fasting blood glucose observed in the DR groups. Systolic blood pressure (SBP) showed slightly higher in NPDR (134.0 ± 19.1 mmHg), and PDR (133.9 ± 18.7 mmHg) groups compared to DM group (132.1 ± 17.1 mmHg). FT3 levels exhibited a graded decline with DR severity (DM: 3.0 ± 0.4 pg/mL, NPDR: 2.9 ± 0.4 pg/mL, PDR: 2.7 ± 0.4 pg/mL, *P* < 0.001). In contrast, sex distribution, smoking history, alcohol consumption, diastolic blood pressure (DBP), TG, TC, HDL-C, LDL-C, and fT4 levels demonstrated no statistically significant differences among groups.

**Table 1:** Baseline characteristics of participants in the cross-sectional study.

	DM (n = 2630)	NPDR (n = 1018)	PDR (n = 55)	*P* value
Age (year)	50.1 ± 12.9	52.0 ± 11.8	53.4 ± 10.7	< 0.001
Sex, n (%)				0.199
Female	1087 (41.3%)	450 (44.2%)	20 (36.4%)	
Male	1543 (58.7%)	568 (55.8%)	35 (63.6%)	
DM duration, n (%)				< 0.001
≥ 10 years	620 (23.6%)	488 (47.9%)	33 (60.0%)	
< 10 years	2010 (76.4%)	530 (52.1%)	22 (40.0%)	
Smoking, n (%)				0.157
Yes	1101 (41.9%)	392 (38.5%)	24 (43.6%)	
No	1525 (58.1%)	626 (61.5%)	31 (56.4%)	
Drinking, n (%)				0.322
Yes	1240 (47.2%)	470 (46.2%)	31 (56.4%)	
No	1385 (52.8%)	548 (53.8%)	24 (43.6%)	
BMI (kg/m^2^)	27.0 ± 4.2	26.7 ± 3.9	26.7 ± 4.2	0.093
Waist circumference (cm)	94.6 ± 11.1	94.9 ± 10.5	96.1 ± 10.8	0.497
Hip circumference (cm)	101.5 ± 8.7	100.4 ± 8.2	100.6 ± 7.5	0.004
SBP (mmHg)	132.1 ± 17.1	134.0 ± 19.1	133.9 ± 18.7	0.025
DBP (mmHg)	80.4 ± 11.5	80.2 ± 12.5	81.2 ± 11.3	0.809
FBG (mmol/L)	9.2 ± 3.8	9.8 ± 4.4	9.3 ± 4.3	< 0.001
Fasting insulin (mU/L)	10.9 (6.9, 16.4)	10.3 (6.0, 15.8)	8.7 (4.5, 15.9)	0.006
HbA1c, n (%)				< 0.001
≥ 7.0%	1885 (72.0%)	896 (88.5%)	46 (85.2%)	
< 7.0%	732 (28.0%)	116 (11.5%)	8 (14.8%)	
TG (mmol/L)	1.5 (1.1, 2.4)	1.5 (1.1, 2.3)	1.7 (1.2, 2.3)	0.548
TC (mmol/L)	4.9 ± 1.4	4.9 ± 1.4	5.1 ± 1.6	0.588
HDL-C (mmol/L)	1.2 ± 0.3	1.2 ± 0.3	1.2 ± 0.3	0.097
LDL-C (mmol/L)	3.1 ± 0.9	3.1 ± 1.0	3.4 ± 1.2	0.132
fT3 (pg/mL)	3.0 ± 0.4	2.9 ± 0.4	2.7 ± 0.4	< 0.001
fT4 (ng/dL)	1.3 (1.2, 1.4)	1.3 (1.2, 1.4)	1.3 (1.2, 1.5)	0.125
TSH (uIU/mL)	1.8 (1.2, 2.7)	1.7 (1.2, 2.6)	1.9 (1.1, 2.9)	0.030
History of medication, n (%)					
Antidiabetic (insulin)	407 (15.5%)	333 (32.8%)	22 (40.0%)	< 0.001
Antidiabetic (non-insulin)	1610 (61.3%)	736 (72.5%)	42 (76.4%)	< 0.001
Antihypertension	867 (33.0%)	390 (38.3%)	20 (36.4%)	0.009
Lipid-lowering	637 (24.2%)	270 (26.5%)	8 (14.5%)	0.074

Continuous variables are expressed as mean ± SD or median (interquartile ranges), while categorical are shown as numbers with percentages.

BMI, body mass index; SBP, systolic blood pressure; DBP, diastolic blood pressure; FBG, fasting blood glucose; HbA1c, glycosylated hemoglobin; TG, triglycerides; TC, total cholesterol; HDL-C, high-density lipoprotein cholesterol; LDL-C, low-density lipoprotein cholesterol; fT3, free triiodothyronine; fT4, free thyroxine; TSH, thyroid stimulating hormone.

### 3.2 Association between baseline fT3 and DR in the cross-sectional study

The association between baseline fT3 levels and the presence of DR was assessed using multivariable logistic regression. As shown in **Table 2**, fT3 levels demonstrated a significant inverse association with the presence of DR (unadjusted OR = 0.53, 95% CI: 0.45–0.62). This inverse relationship persisted after comprehensive adjustment for age, sex, smoking status, alcohol consumption, DM duration, BMI, hip circumference, SBP, HbA1c, HDL-C, TSH and history of medication, yielding an adjusted OR of 0.58 (95% CI: 0.48–0.70). Furthermore, after adjustment for the same covariates, higher fT3 levels were significantly associated with lower odds of NPDR (adjusted OR = 0.61, 95% CI: 0.50-0.74) and PDR (adjusted OR = 0.24, 95% CI: 0.13-0.44).

**Table 2:** Independent effect of fT3 levels on the presence and severity of DR.

	Crude		Adjusted	
	OR (95% CI)	*P* value	OR (95% CI)	*P* value
The presence of DR	0.53 (0.45, 0.62)	< 0.001	0.58 (0.48, 0.70)	< 0.001
The severity of DR				
NPDR	0.55 (0.47, 0.65)	< 0.001	0.61 (0.50, 0.74)	< 0.001
PDR	0.28 (0.16, 0.48)	< 0.001	0.24 (0.13, 0.44)	< 0.001

The fully adjusted logistic models were controlled for age, sex, smoking status, alcohol consumption, DM duration, BMI, hip circumference, SBP, HbA1c, HDL-C, TSH, history of medication.

BMI, body mass index; SBP, systolic blood pressure; HbA1c, glycosylated hemoglobin; HDL-C, high-density lipoprotein cholesterol; fT3, free triiodothyronine; TSH, thyroid stimulating hormone.

### 3.3 Association between baseline fT3 and DR onset or progression

**Figure 1:**
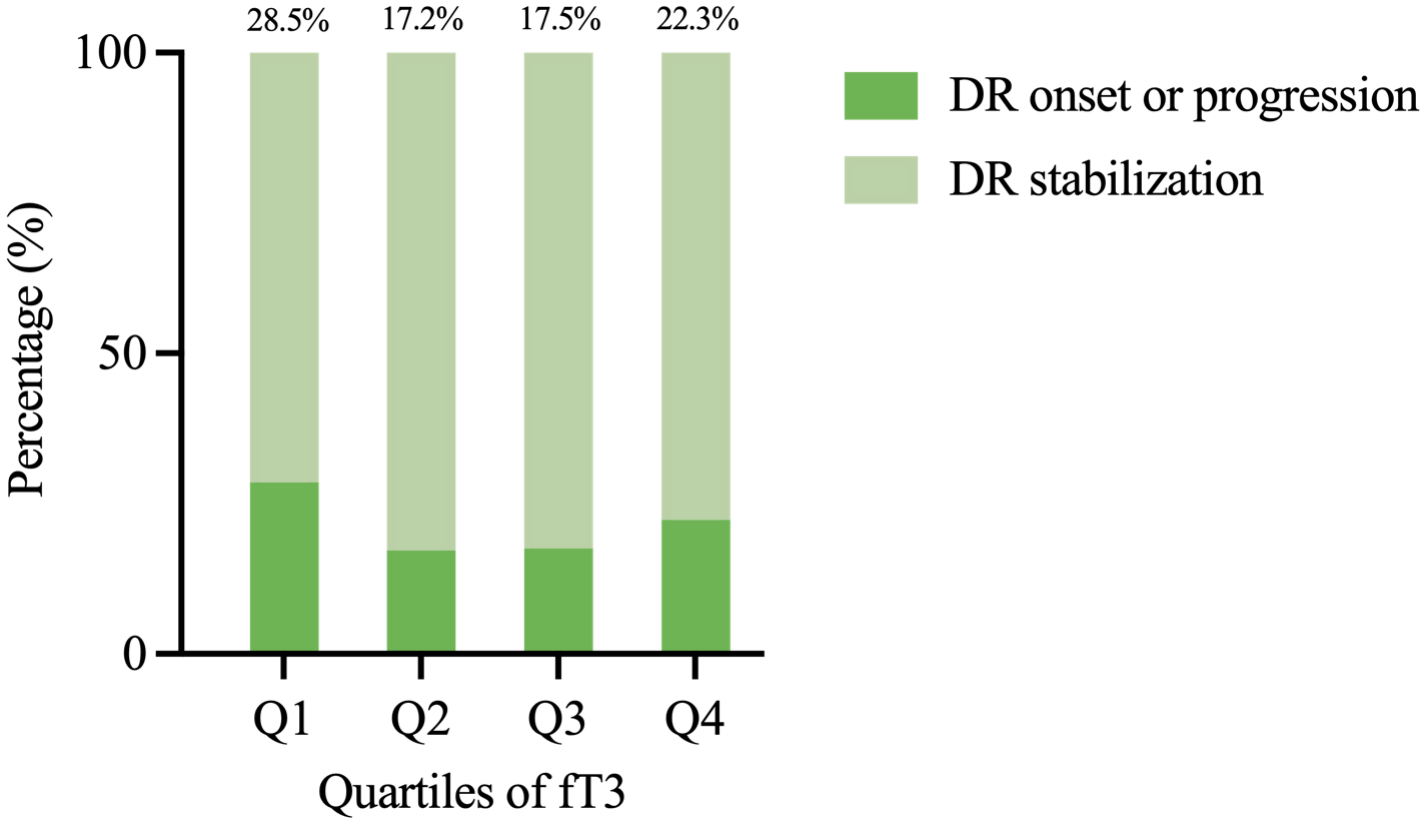
Prevalence of DR onset or progression by fT3 quartiles.

In the retrospective cohort study, 1476 patients were stratified into quartiles by baseline fT3 levels (Q1: ≤ 2.70 pg/ml, Q2: 2.70-2.98 pg/ml, Q3: 2.98-3.27 pg/ml, Q4: > 3.27 pg/ml). The baseline characteristics of the participants in the cohort analysis were shown in **Supplementary Table 1**. During a median follow-up duration of 22.4 months (interquartile range 12.4-33.8 months), a total of 314 patients (21.3%) developed DR or DR progression. Compared with the first fT3 quartile group (Q1), the risk of DR onset or progression was significantly decreased in Q2, Q3 and Q4 (**Figure 1**). Univariate logistic regression identified several potential risk factors for DR onset or progression (**Supplementary Table 2**). In multivariable analysis adjusted for age, sex, DBP, SBP, smoking status, alcohol consumption, DM duration, HbA1c, BMI, TG and history of medication, higher fT3 levels showed an inverse association with DR onset or progression risk. Compared with Q1, the odds of DR onset or progression decreased by 43% (95%CI: 0.39-0.82, *P* = 0.003) in Q2, 38% in Q3 (95%CI: 0.43-0.91, *P* = 0.013), and 19% in Q4 (95%CI: 0.56-1.19, *P* = 0.291) (**Table 3**). These results suggested a potential protective effect of moderate fT3 levels (Q2-Q3), whereas the association in Q4 was not statistically significant (**Table 3**).

**Table 3:** Risk of onset or progression of DR for baseline fT3 levels.

	Crude		Adjusted	
	OR (95% CI)	*P* value	OR (95% CI)	*P* value
Overall				
Q1	Ref	Ref	Ref	Ref
Q2	0.52 (0.37, 0.74)	< 0.001	0.57 (0.39, 0.82)	0.003
Q3	0.53 (0.38, 0.75)	< 0.001	0.62 (0.43, 0.91)	0.013
Q4	0.72 (0.52, 1.01)	0.059	0.81 (0.56, 1.19)	0.291

The fully adjusted logistic models were controlled for age, sex, DBP, SBP, smoking status, alcohol consumption, DM duration, HbA1c, BMI, TG and history of medication. Notably, moderate fT3 levels (Q2 and Q3) were associated with a lower risk of DR onset or progression.

BMI, body mass index; SBP, systolic blood pressure; DBP, diastolic blood pressure; LDL-C, low-density lipoprotein cholesterol; fT3, free triiodothyronine.

### 3.4 Subgroup analysis of fT3 and DR onset or progression

**Figure 2:**
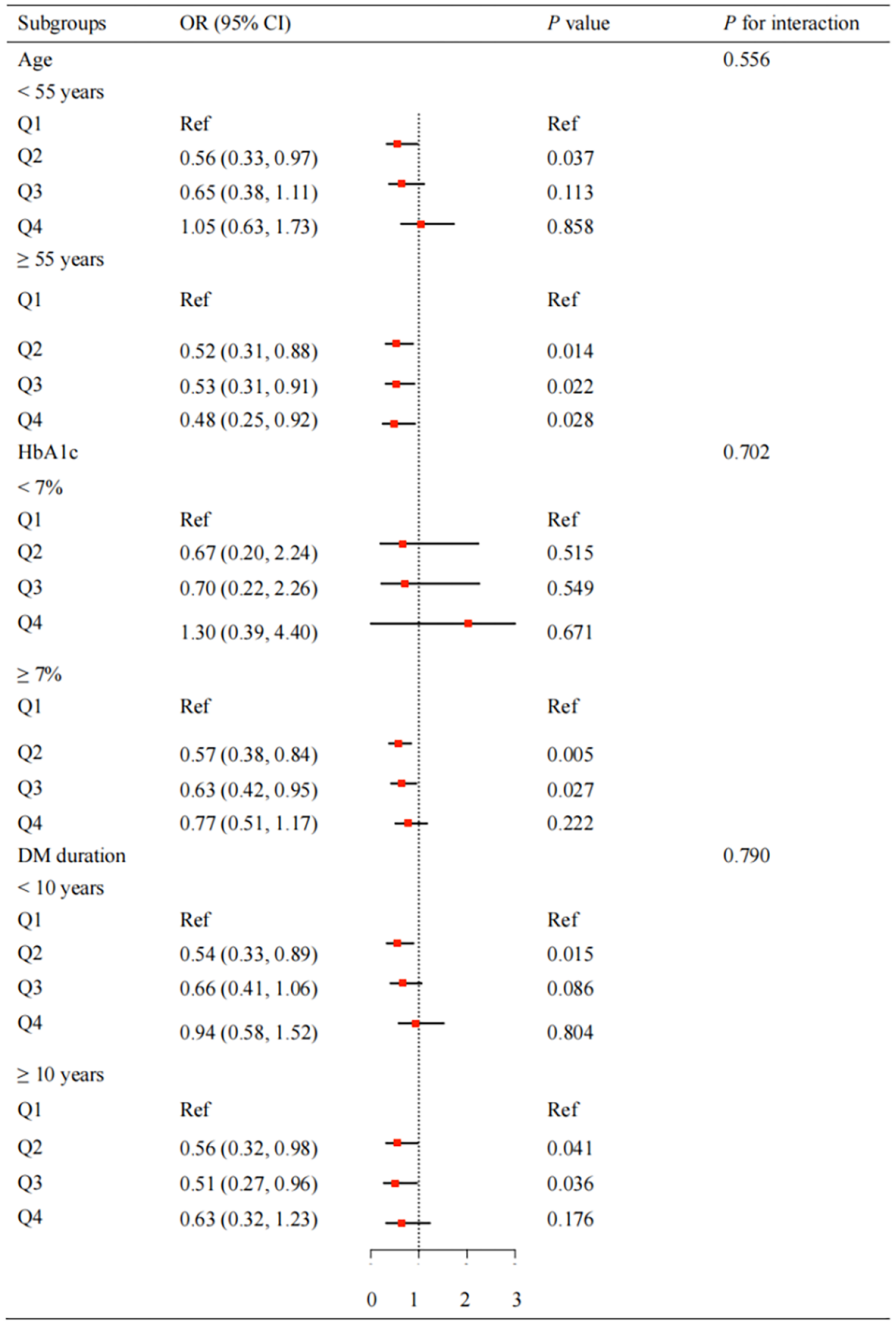
Subgroup analysis of fT3 and DR onset or progression. The fully adjusted logistic models were controlled for age, sex, DBP, SBP, smoking history, drinking history, DM duration, BMI, HbA1c, TG and history of medication. BMI, body mass index; SBP, systolic blood pressure; DBP, diastolic blood pressure; HbA1c, glycosylated hemoglobin; L TG, triglycerides; fT3, free triiodothyronine.

To further explore the association between fT3 levels and the risk of DR onset or progression, we conducted subgroup and interaction analysis stratified by age (< 55 or ≥ 55 years), HbA1c (< 7% or ≥ 7%) and DM duration (< 10 or ≥ 10 years) (**Supplementary Table 3**). As shown in **Figure 2**, among participants with poor glycemic control (HbA1c ≥ 7%), the inverse association between fT3 levels and DR onset or progression remained statistically significant in Q2 (43% reduction, OR = 0.57, 95% CI: 0.38-0.84, *P* = 0.005) and Q3 (37% reduction, OR = 0.63, 95% CI: 0.42-0.95, *P* = 0.027). Similarly, in patients with longer diabetes duration (≥ 10 years), Q2-Q3 showed pronounced protective effects (Q2: 44% reduction, OR = 0.56, 95% CI: 0.32-0.98, *P* = 0.041; Q3: 49% reduction, OR = 0.51, 95% CI: 0.27-0.96, *P* = 0.036). Notably, in older patients (≥ 55 years), the benefit extended across a wider range of fT3 levels (Q2: OR = 0.52, 95% CI: 0.31-0.88, *P* = 0.014; Q3: OR = 0.53, 95% CI: 0.31-0.91, *P* = 0.022; Q4: OR = 0.48, 95% CI: 0.25-0.92, *P* = 0.028).

### 3.5 MR analysis reveals a protective effect of fT3 on NPDR

**Figure 3:**
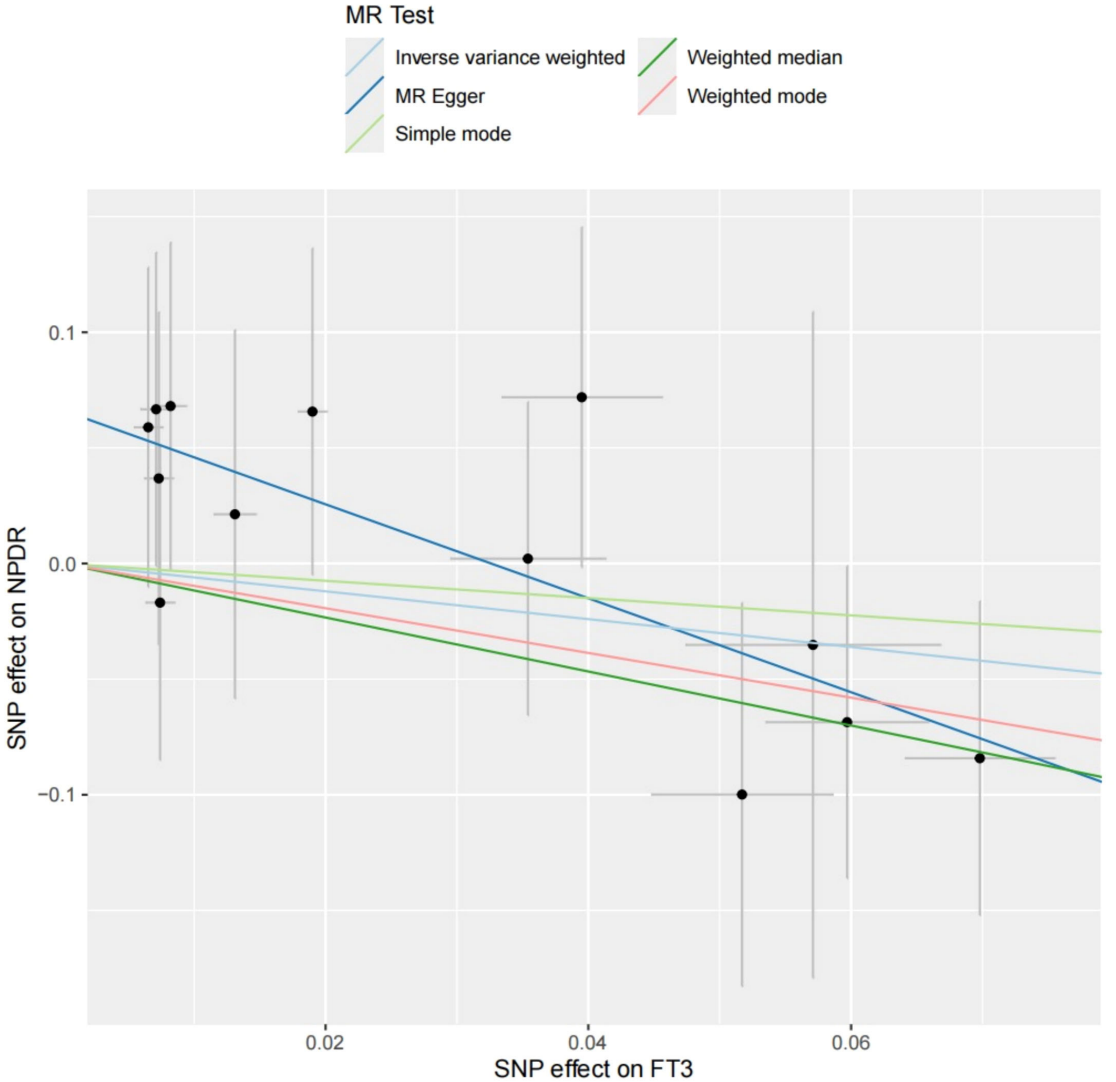
Scatter plot of MR analysis of the association between fT3 and NPDR. Each point represents an individual SNP, with the x-axis showing its effect size on the exposure and the y-axis its effect size on the outcome.

This MR analysis assessed the causal effects of genetically predicted fT3 levels on NPDR and PDR using 13 associated SNPs as instrumental variables. For NPDR, the MR-Egger method indicated a significant inverse association between fT3 and disease risk (MR Egger, OR = 0.131, 95% CI: 0.023-0.755, *P* = 0.044) (**Table 4**). Although other methods, including inverse variance weighted (IVW) and weighted median, were not statistically significant, the point estimates for all methods were consistently oriented toward a protective direction. Sensitivity analyses showed no significant horizontal pleiotropy (*P* = 0.06) or heterogeneity (MR Egger’s test, Q-statistic *P* = 0.98), suggesting that the findings are robust and reliable (**Supplementary Tables 6, 7**, **Supplementary Figures 2–4**). The scatter plot further confirmed a negative trend across MR methods (**Figure 3**). In contrast, no significant associations were observed between fT3 and PDR risk across all MR methods (MR-Egger OR = 0.658, 95% CI: 0.235-1.844, *P* = 0.442; IVW OR = 0.977, 95% CI: 0.507-1.881, *P* = 0.944), with effect estimates close to null and wide confidence intervals (**Supplementary Table 4**). The scatter plot showed inconsistent directions among SNPs (**Supplementary Figure 5**). These findings suggested a potential protective effect of higher fT3 levels on NPDR, but not in advanced PDR. Further research is needed to validate these subtype-specific effects.

**Table 4:** Two-sample MR estimates of associations between fT3 levels and NPDR using various analysis methods.

Exposure	nsnp	Method	P value	OR (95% CI)
fT3 || NPDR	13	MR Egger	0.044	0.131 (0.023, 0.755)
	13	Weighted median	0.102	0.311(0.077, 1.261)
	13	Inverse variance weighted	0.289	0.548 (0.180, 1.668)
	13	Simple mode	0.719	0.688 (0.095, 5.012)
	13	Weighted mode	0.172	0.380 (0.103, 1.404)

## 4 Discussion

Our study extends existing evidence by elucidating cross-sectional, longitudinal associations and MR analysis between fT3 levels and DR, with a novel emphasis on the inversed association with both NPDR and PDR. While prior studies research has largely focused on cross-sectional associations, our findings incorporate longitudinal data and MR analysis, offering stronger support for a potential positive effect of fT3 on DR.

Previous studies have primarily linked thyroid dysfunction to DR risk through cross-sectional designs. Zahra Heidari et al. reported a 7.7% increase in the prevalence of subclinical hypothyroidism among DR patients (13), while Wang et al. identified elevated TSH levels within the euthyroid ranges as an independent risk factor for DR in 2740 T2DM patients (19). Another cross-sectional study revealed that fT3 levels within the normal range were negatively associated with DR in euthyroid patients with T2DM (12). Our findings align with these observations but advance the field by demonstrating that higher fT3 levels confer stronger protection against PDR than NPDR, possibly due to differential roles of thyroid hormones in early microvascular leakage versus advanced neovascularization. Our MR results extend beyond cross-sectional correlations by providing genetic evidence consistent with a positive effect of fT3 on NPDR. Furthermore, compared with patients with lower fT3 levels, those with moderate fT3 levels exhibited a significantly reduced risk of DR onset or progression during the 22.4-month follow-up. This pattern suggests a non-linear relationship between fT3 levels and DR risk, where excessively low or high fT3 may be detrimental, and only moderate levels are protective. One potential explanation is that moderate fT3 concentrations may reflect an optimal metabolic and hormonal state that supports vascular stability without triggering adverse effects such as increased oxidative stress or sympathetic activation, which may occur at higher fT3 levels. Thyroid hormones influence endothelial nitric oxide production, lipid metabolism, and mitochondrial function—processes that, when balanced, contribute to the protection of the retinal microvasculature from damage. However, supraphysiological fT3 levels may enhance metabolic rate excessively, leading to increased oxidative burden and endothelial dysfunction, offsetting the protective effects. Notably, in older patients (≥ 55 years), the benefit extended across a wider range of fT3 levels (Q2-Q4). We hypothesize that this reflects an age-related elevation in the threshold of thyroid hormone requirement for retinal protection. This observation underscores the potential for personalized, age-specific considerations when evaluating thyroid function in the context of diabetic complications.

Several plausible mechanisms may underlie this protective association. As a key metabolic regulator, fT3 influences endothelial function, oxidative stress, and glucose metabolism—core pathways in DR pathogenesis. Moderate fT3 levels may thus confer protection by stabilizing retinal vasculature and mitigating insulin resistance and systemic inflammation, consistent with clinical observations linking higher fT3 to improved metabolic profiles in T2DM (12, 20). Moreover, thyroid hormones regulate mitochondrial biogenesis and oxidative phosphorylation through pathways involving peroxisome proliferator-activated receptor gamma coactivator 1-alpha (PGC-1α) and mitochondrial fusion proteins (21). Experimental models demonstrate that retinal T3 deficiency exacerbates diabetic mitochondrial damage, leading to ATP depletion, elevated reactive oxygen species (ROS), and accelerated apoptosis of vascular cells (21, 22). Additionally, TSH receptor activation under hyperglycemia directly induces apoptosis in human retinal pericytes (23), suggesting dual pathways linking thyroid axis dysregulation to DR pathogenesis.

Our study addresses key limitations of prior research by combining severity-stratified analysis with longitudinal assessment of DR onset or progression. The graded inverse association between fT3 and DR severity suggests that thyroid function monitoring may refine DR risk stratification, especially among patients with poor glycemic control, advanced age, or longer DM duration. However, several important limitations must be considered. First, the single-center, retrospective design and relatively short follow-up period may affect the generalizability of our findings (particularly across ethnic groups) and limit our ability to establish long-term causal relationships. Ethnic variability in DR phenotypic characteristics, and environmental modifiers may lead to heterogeneous thyroid-DR associations between Asian and Caucasian population (24, 25). Second, although we comprehensively adjusted for metabolic confounders, residual confounding from unmeasured factors such as thyroid autoantibodies cannot be excluded. In addition, our findings await external validation in independent, multi-ethnic cohorts to confirm their broader applicability. Despite these limitations, our analyses strengthen the plausibility of a thyroid–retinopathy link and highlight fT3 as a potential biomarker worthy of further prospective investigation.

In conclusion, our study demonstrated an inverse association between fT3 levels and the risk of both NPDR and PDR. Moderate fT3 levels were associated with a lower risk of DR onset or progression, particularly among patients with suboptimal glycemic control (HbA1c ≥7%) or longer diabetes duration (≥ 10 years). In older patients (≥ 55years), even relatively higher fT3 levels may be protective. Additionally, MR analysis suggested a potential protective effect of elevated fT3 levels on the risk of NPDR, which was significant only in the MR Egger model. These findings suggest the potential clinical importance of monitoring fT3 levels in high-risk diabetic populations. Future studies should incorporate serial fT3 measurements in diverse, multiethnic cohorts to elucidate the dynamic interplay between thyroid function and DR progression.

## Data Availability

The datasets used in the study are available from the corresponding author on reasonable request. Requests to access the datasets should be directed to JK, kejing@ccmu.edu.cn.
