# Supplementary material for: Association between free triiodothyronine and diabetic retinopathy: insights from a longitudinal cohort study and Mendelian randomization
Source: Front Endocrinol (Lausanne). 2025 Dec 17;16:1677122. doi: 10.3389/fendo.2025.1677122 (PMC12753430; doi:10.3389/fendo.2025.1677122)
Supplement: Supplementary file 1 [file DataSheet1.docx]

**Supplemental Figure legends**

**Supplemental Figure 1.** Supplementary Figure 1 Flow chart of participants selection.

**Supplemental Figure 2.** Leave-One-Out sensitivity analysis for fT3 and NPDR association.

**Supplemental Figure 3.** Forest plot of individual SNP and overall IVW estimates for fT3 on NPDR risk.

**Supplemental Figure 4.** Funnel plot assessing pleiotropy in MR analysis of fT3 and NPDR.

**Supplemental Figure 5.** Scatter plot of MR analysis of the association between fT3 and PDR.

**Supplemental Figure 6.** Leave-One-Out sensitivity analysis for the association between fT3 and PDR.

**Supplemental Figure 7.** Forest plot of individual SNP and overall IVW estimates for fT3 on PDR risk.

**Supplemental Figure 8.** Funnel plot assessing pleiotropy in MR analysis of fT3 and PDR.

**
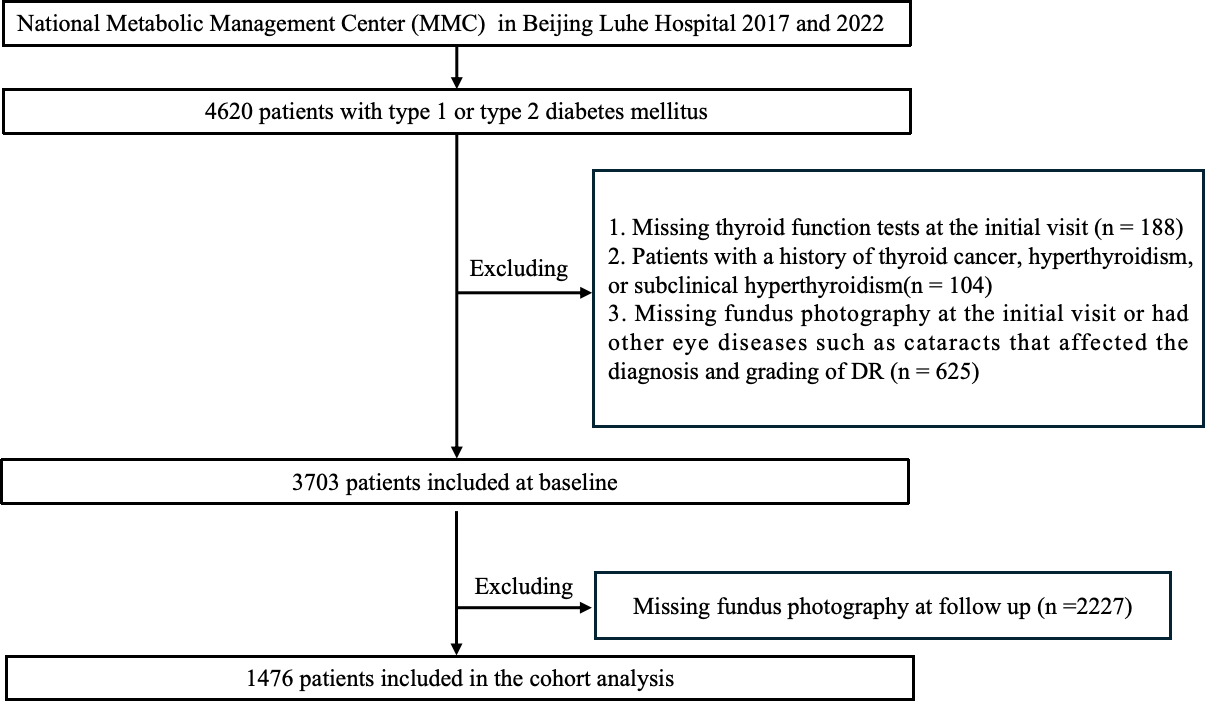
**

**Supplemental Figure 1 Flow chart of participants selection.**

**
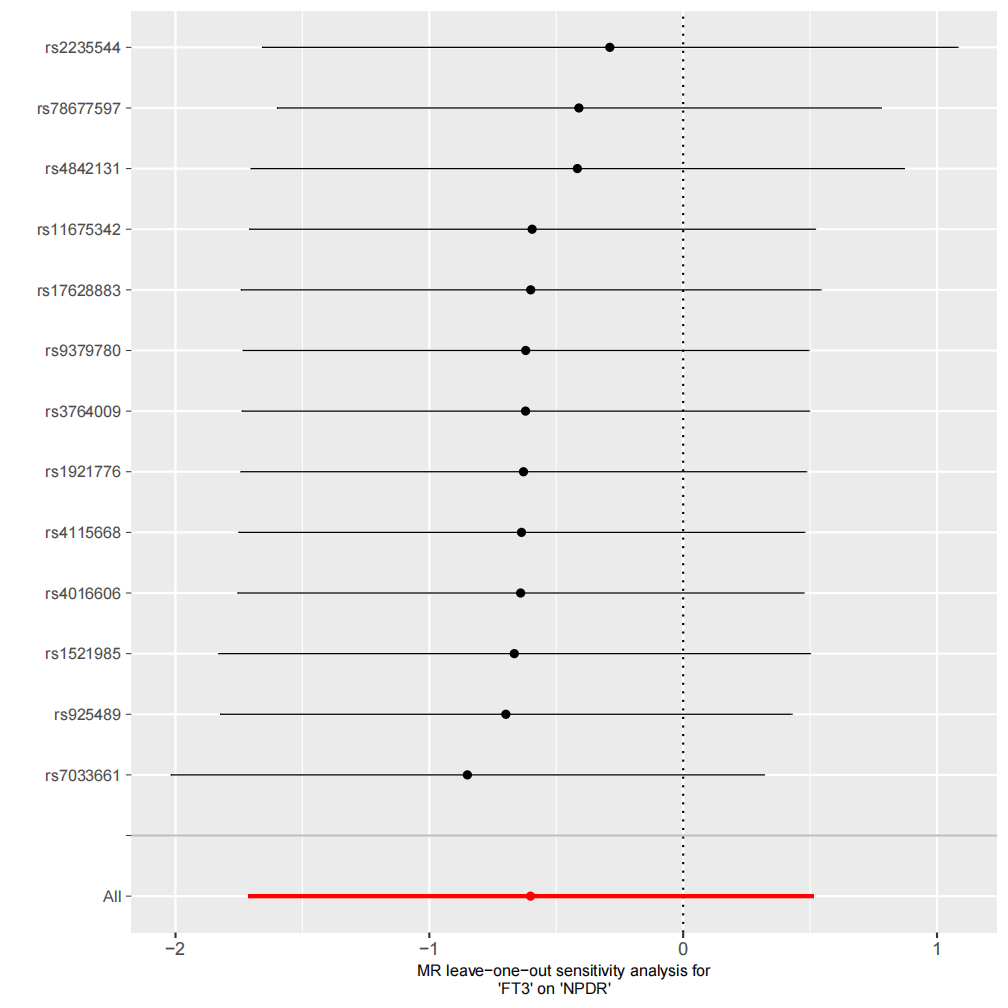
 Supplemental Figure 2 Leave-One-Out sensitivity analysis for fT3 and NPDR association.** Each point represents the causal estimate obtained by sequentially excluding one single-nucleotide polymorphism (SNP) at a time.

**
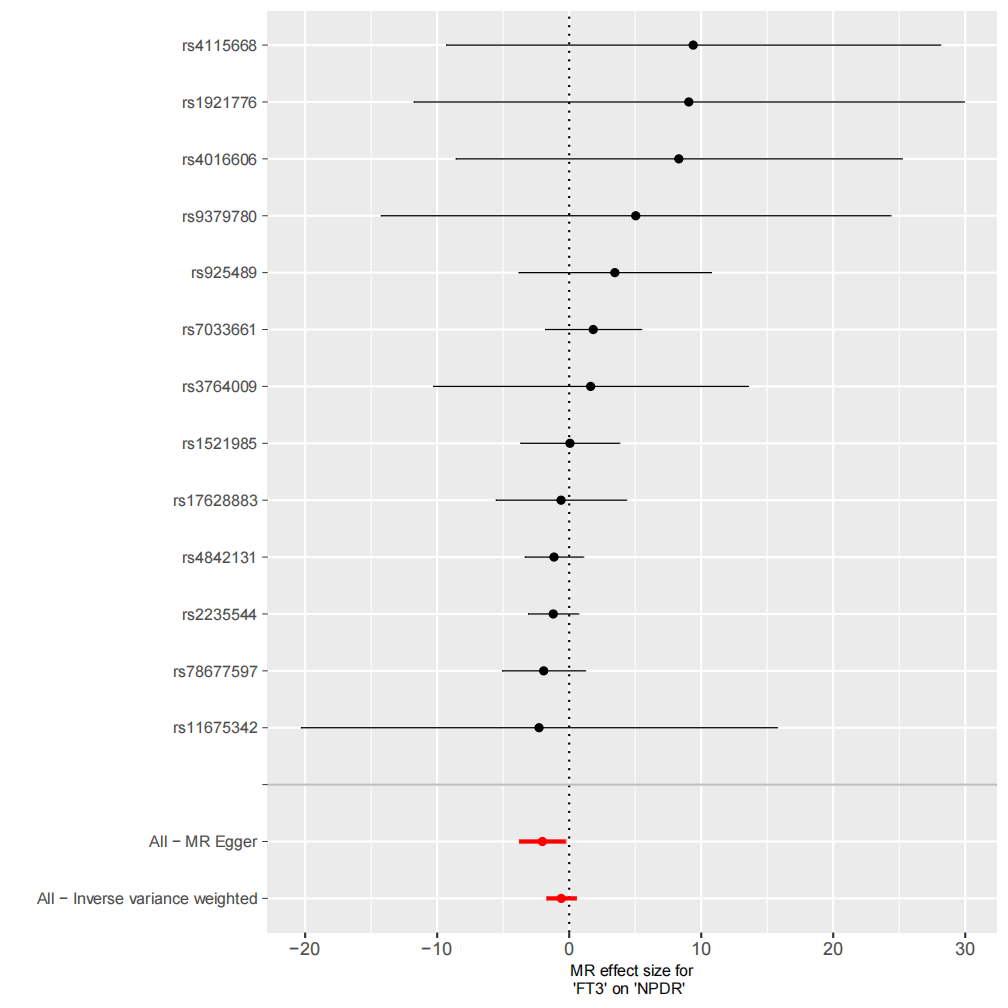
**

**Supplemental Figure 3 Forest plot of individual SNP and overall IVW estimates for fT3 on NPDR risk.**

**
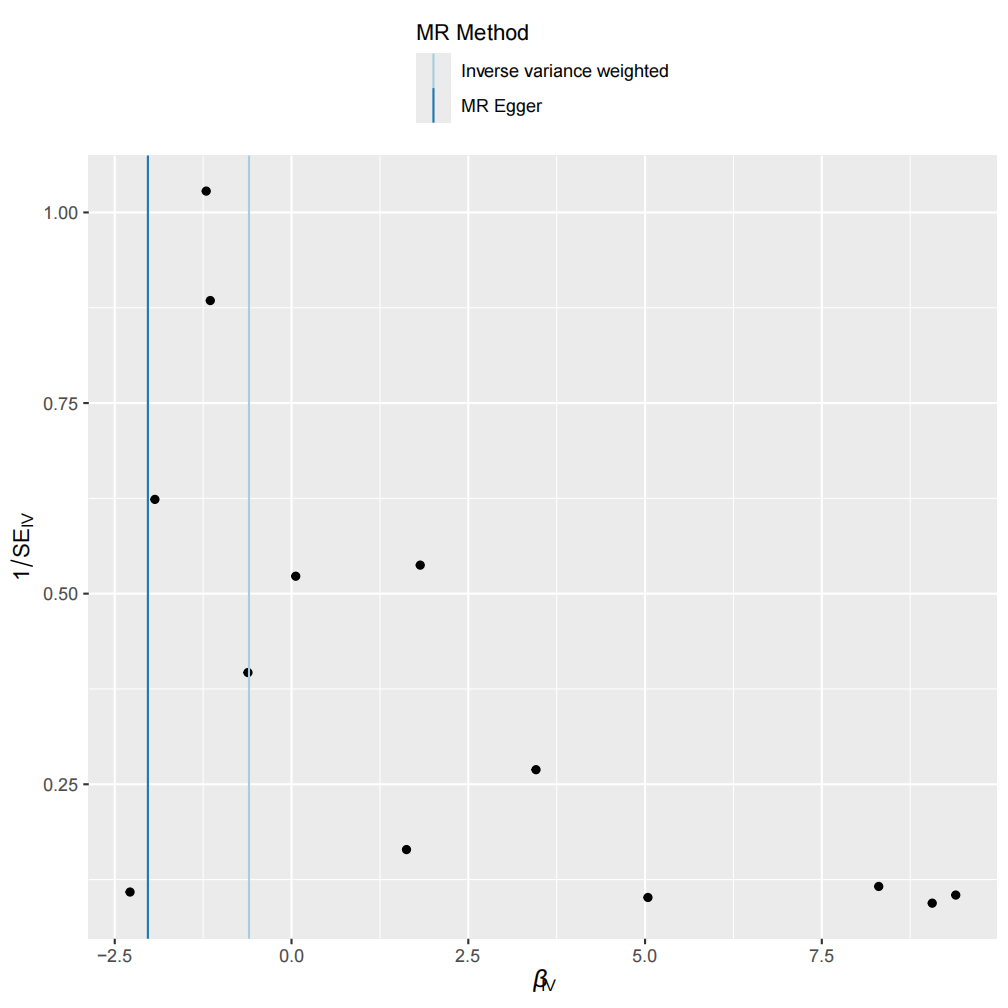
**

**Supplemental Figure 4 Funnel plot assessing pleiotropy in MR analysis of fT3 and NPDR.**


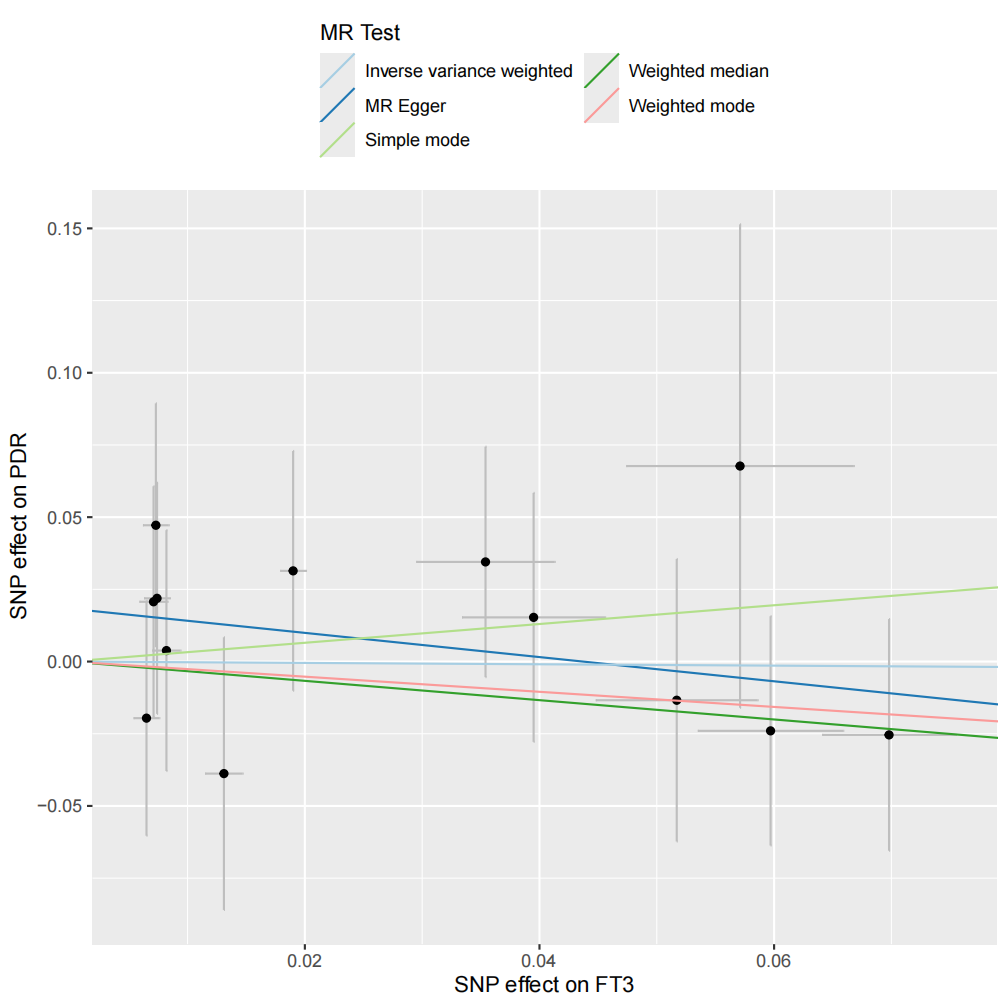


**Supplemental Figure 5** **Scatter plot of MR analysis of the association between fT3 and PDR.** Each point represents an individual SNP, with the x-axis showing its effect size on the exposure and the y-axis showing its effect size on the outcome.


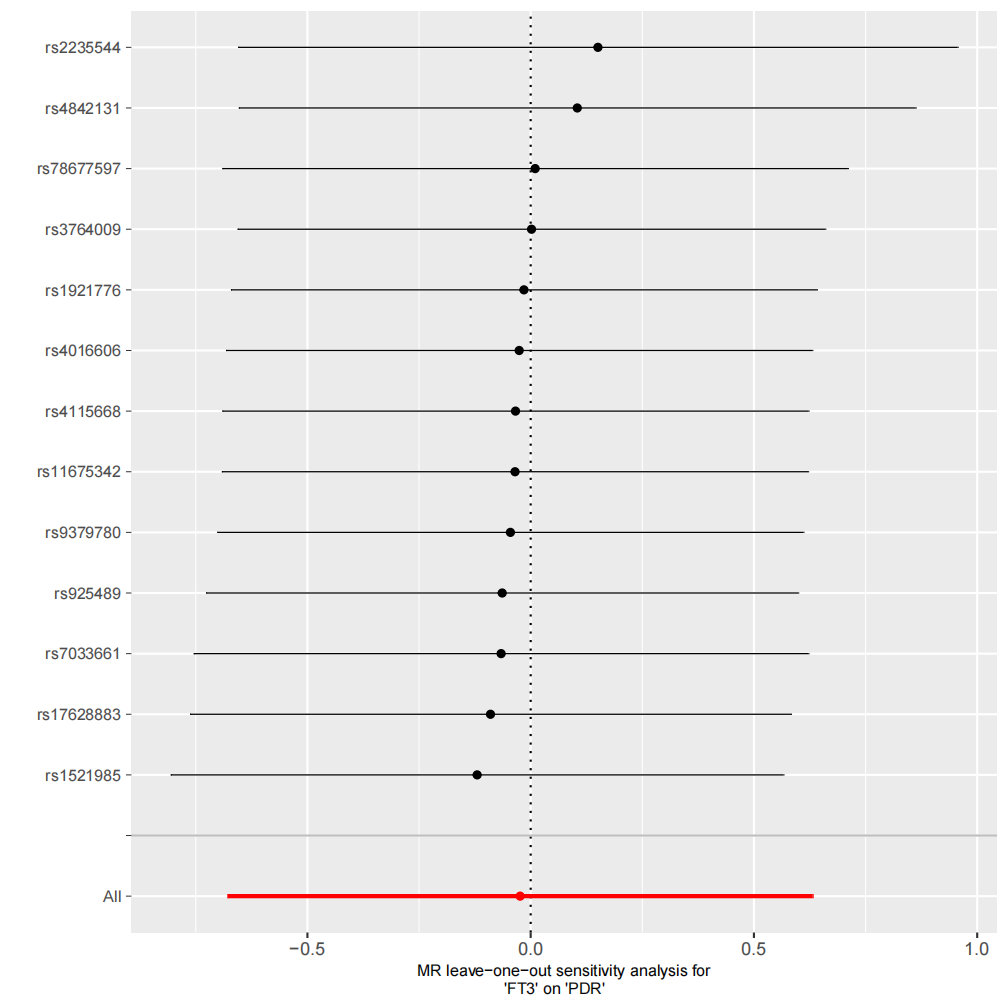


**Supplemental Figure 6 Leave-One-Out sensitivity analysis for the association between fT3 and PDR.** Each point represents the causal estimate obtained by sequentially excluding one single-nucleotide polymorphism (SNP) at a time.


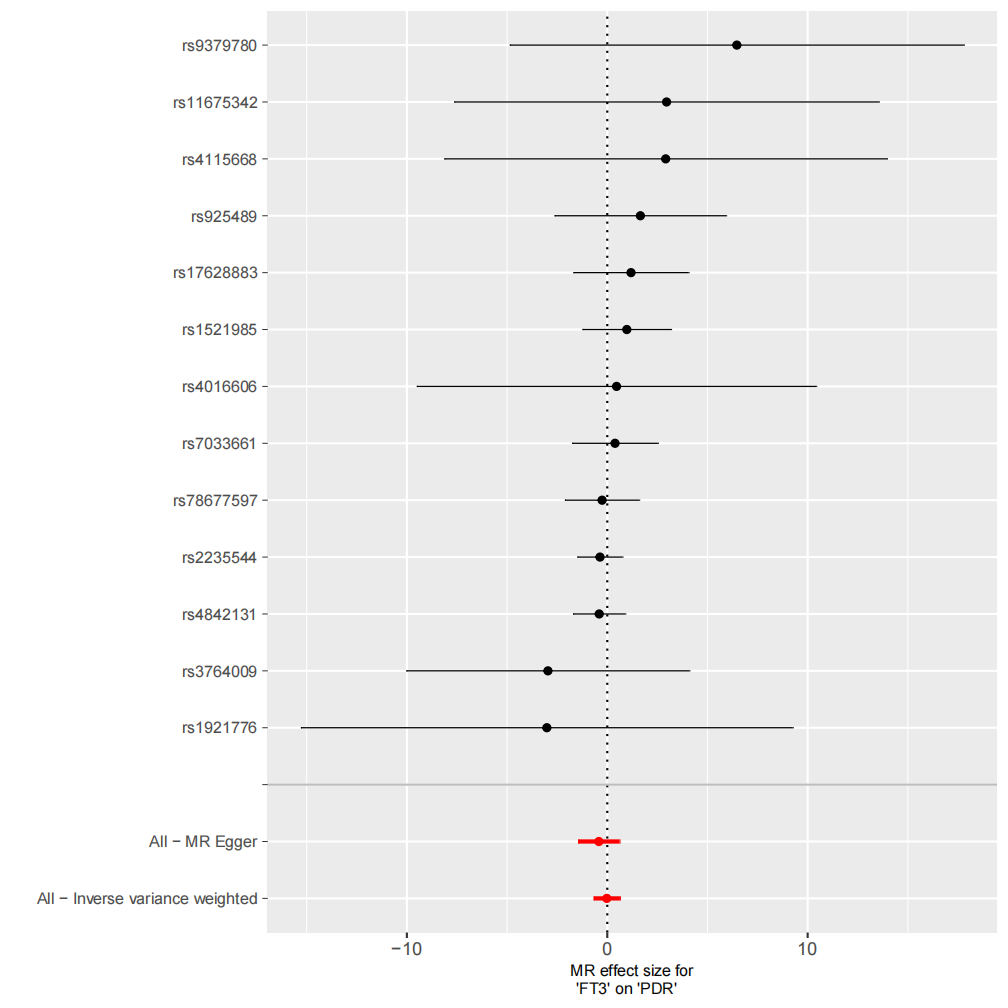


**Supplemental Figure 7 Forest plot of individual SNP and overall IVW estimates for fT3 on PDR risk.**

**Supplemental Figure
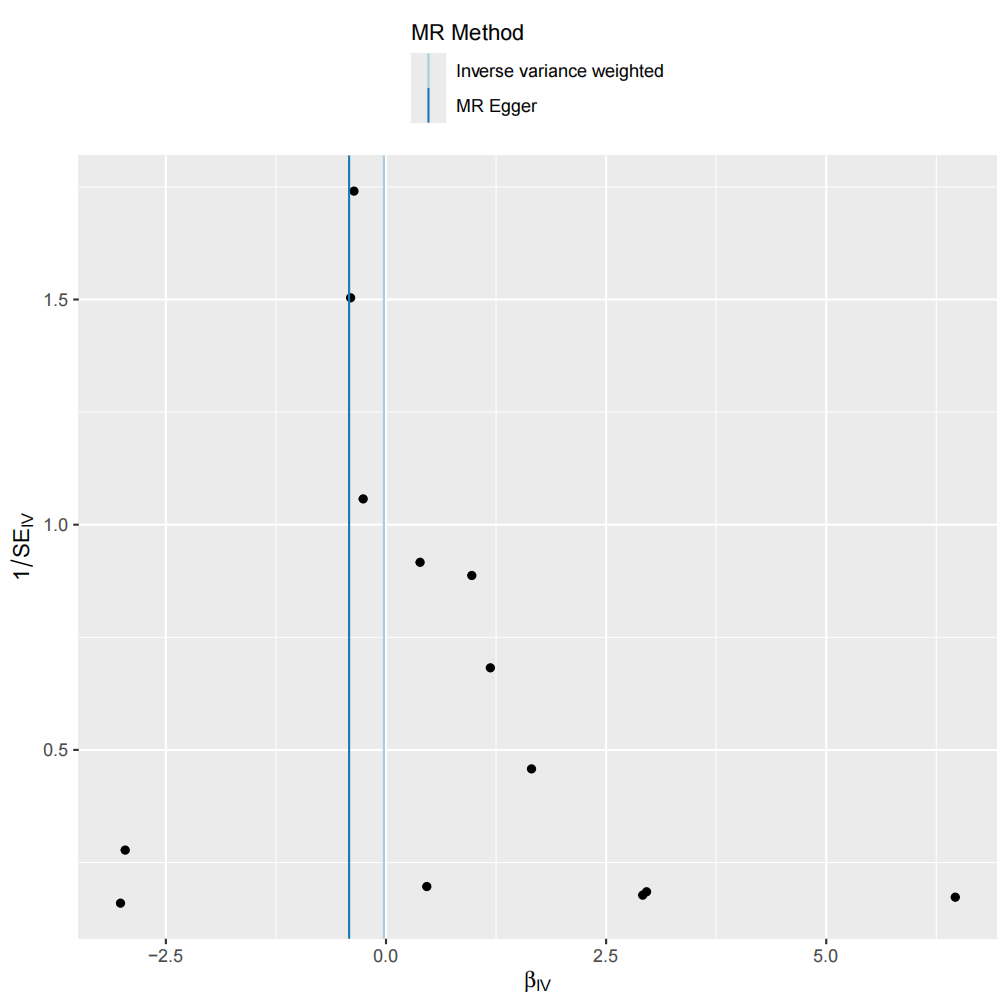
8** **Funnel plot assessing pleiotropy in MR analysis of fT3 and PDR.**
